# Promoter Methylation-Mediated Silencing of β-Catenin Enhances Invasiveness of Non-Small Cell Lung Cancer and Predicts Adverse Prognosis

**DOI:** 10.1371/journal.pone.0112258

**Published:** 2014-11-14

**Authors:** Yuan Miao, Liang Wang, Xiupeng Zhang, Xiaohan Xu, Guiyang Jiang, Chuifeng Fan, Yang Liu, Xuyong Lin, Juanhan Yu, Yong Zhang, Enhua Wang

**Affiliations:** 1 Department of Pathology, The First Hospital and College of Basic Medical Sciences of China Medical University, Shenyang, China; 2 96K Seven-year Program of Medicine, China Medical University, Shenyang, China; University of Parma, Italy

## Abstract

β-Catenin plays dual role in adhesion complex formation and the Wnt signaling pathway. Although β-catenin expression appears to be upregulated and Wnt signaling pathway is activated in the majority of cancers, its expression level seems to be lost in non-small cell lung cancer (NSCLC). We previously reported that the promoter of β-catenin was hypermethylated in two NSCLC cell lines. In the current study, we expanded our analysis for the methylation status of β-catenin promoter region and its protein expression in seven NSCLC cell lines and a series of 143 cases of primary human lung cancer with adjacent non-neoplastic tissues. Quantitative methylation specific PCR (qMSP) analysis showed methylation of β-catenin promoter region in five NSCLC cell lines, with increased β-catenin protein levels upon 5′-Aza-2′-deoxycytidine (5-aza-dC) treatment. The methylation status in SPC (methylated) and A549 (unmethylated) was confirmed by bisulfite sequencing PCR. 5-Aza-dC treatment inhibited invasiveness of SPC but not A549. Immunofluorescence analysis showed membranous β-catenin expression was lost in SPC and could be re-established by 5-aza-dC, while Wnt3a treatment led to nuclear translocation of β-catenin in both SPC and A549. Dual-luciferase assays indicated that 5-aza-dC treatment caused no significant increase in Wnt signaling activity compared with Wnt3a treatment. The effect of demethylation agent in SPC can be reversed by β-catenin depletion but not E-cadherin depletion which indicated that the methylation mediated β-catenin silencing might enhance NSCLC invasion and metastasis in an E-cadherin independent manner. Subsequent immunohistochemistry results further confirmed that β-catenin promoter hypermethylation correlated with loss of immunoreactive protein expression, positive lymph node metastasis, high TNM stage and poor prognosis. The present study implicates β-catenin promoter hypermethylation in the mechanism of epigenetic changes underlying NSCLC metastasis and progression, thus indicating the potential of β-catenin as a novel epigenetic target for the treatment of NSCLC patients.

## Introduction

Lung cancer remains a major clinical challenge because of its poor prognosis and limited treatment options [1]. The high mortality of lung cancer is largely attributable to failure of early diagnosis and metastasis frequently observed at the time of diagnosis [2]. Analysis of the biological changes underlying the pathogenesis of this malignancy, identification in the early stages, and the prevention of metastasis, therefore, may be the primary options for improving the overall dismal prognosis of this cancer.

Tumor invasion and metastasis occur when tumor cells gain the ability to detach from the primary tumor and enter into surrounding tissue or lymphovascular channels, a process that is critically dependent on the disruption of adhesion junctions between tumor cells [3]. β-Catenin is especially interesting during this process since it not only participates in this adhesion complex, but also an integral part of the Wnt signaling pathway [4]–[6]. A vast majority of cancers including colorectal, hepatocellular, thyroid, and ovarian cancers, harbor β-catenin mutations and changes in other genes, such as the APC gene, which leads to nuclear accumulation of β-catenin and activation of the Wnt signaling pathway [4], [7], [8]. However, evidence indicates that nuclear expression of β-catenin is rare and Wnt signaling activity is downregulated in lung cancer [5], [9]–[11]. A growing number of studies have described and analyzed the association between loss of β-catenin expression and clinicopathological parameters in lung cancer patients [9], [11]. Nevertheless, the underlying mechanisms remain controversial. A recent report showed that the level of β-catenin protein was not affected by E-cadherin depletion [12]. Therefore, there must be other mechanisms responsible for downregulated β-catenin expression in NSCLC.

Aberrant promoter methylation could result in gene silencing in various malignant tumors including lung cancer [13], [14]. Although the Wnt signaling was activated and β-catenin expression was upregulated in primary gastric carcinoma, Ebert *et al.* showed that the promoter of β-catenin was hypermethylated which led to loss of β-catenin expression in the cell line derived from liver metastasis of gastric cancer but not those primary cancers [15]. Taken together, we speculated that methylation of β-catenin promoters may play a role in regulating the invasiveness of cancer cells. Our most recent report found that hypermethylation of β-catenin promoters were seen in two primary NSCLC cell lines, which differed from that in gastric cancer [16]. Since the activities of Wnt signaling pathway was lower in NSCLC than what in other cancers, we hypothesized that hypermethylation of β-catenin promoters in NSCLC may account for the high metastasis frequency and poor prognosis in NSCLC patients.

Here, we studied the impact of the β-catenin promoter methylation statuson tumor progression and invasion, by utilizing both lung cancer cell lines and patients samples as our experimental models.

## Materials and Methods

### Human tissue samples and cell lines

This study was conducted with the approval of the institutional review board at China Medical University (Shenyang, China). Written consent was given by the participants for their information to be stored in the hospital database for their specimens to be used in this study. All clinical investigation has been conducted according to the principles expressed in the Declaration of Helsinki. One hundred and forty-three cases of primary human lung cancer with corresponding adjacent non-neoplastic tissues who underwent surgical resection with curative intent, were collected from the First Affiliated Hospital of China Medical University between October 2004 and July 2006. Patients' survival was defined as the time from the day of surgery to the end of follow-up or day of death due to recurrence or metastasis. None of the patients received radiotherapy or chemotherapy before surgical resection, and all patients received routine chemotherapy after surgery. The study patient population has been reported in our previous literature [17]. The histological diagnosis and differentiation grade were evaluated in hematoxylin and eosin stained sections, according to the 2004 WHO classification guidelines [18]. All 143 specimens were reevaluated with respect to the histological subtype, differentiation, and tumor stage. Tumor staging was determined according to the seventh edition of the International Union against Cancer (UICC) TNM Staging System for Lung Cancer [19]. The samples included 44 cases of squamous cell lung carcinoma and 99 cases of lung adenocarcinoma. Fifty-one tumors were highly differentiated and 92 tumors were moderately or poorly differentiated. Lymph node metastases were present in 70 cases and absent in 73 cases. The tumors included 81 cases of stage I–II disease and 62 cases of stage III_A_–III_B_ disease.

Seven NSCLC cell lines, commonly used in our lab, were selected for cell experiments. The HBE, A549 and H1299 cell lines were obtained from the American Type Culture Collection (Manassas, VA, USA). The PG-BE1 (BE1), PG-LH7 (LH7), SPC-A-1 (SPC), and LTEP-A-2 (LTE) cell lines were obtained from Shanghai Cell Bank (Shanghai, China). All cells were cultured in RPMI 1640 (Invitrogen, Carlsbad, CA, USA) containing 10% fetal calf serum (Invitrogen), 100 IU/ml penicillin (Sigma, St. Louis, MO, USA), and 100 µg/ml streptomycin (Sigma). The cells were grown in sterile culture dishes and passaged every 2 days using 0.25% trypsin (Invitrogen).

### DNA extraction and quantitative real-time methylation-specific polymerase chain reaction

Genomic DNA was extracted from cell lines and 10-µm-thick sections of 10% neutral formalin-fixed, paraffin-embedded tumor tissue blocks using the QIAamp DNA Mini Kit (QIAGEN, Hilden, Germany). Genomic DNA was treated with sodium bisulfite using an EZ DNA methylation kit (D5005, Zymo Research, Orange, CA, USA). Methylation-specific real-time polymerase chain reaction (PCR) assays were performed in the ABI 7900HT Fast Real-time PCR system (Applied Biosystems, Foster City, CA, USA). Primer pairs were as follows: β-catenin forward, 5′-GGAAAGGCGCGTCGAGT-3′ and reverse, 5′-TCCCCTATCCCAAACCCG-3′, with the TaqMan probe 5′-6FAM- CGCGCGTTTCCCGAACCG-TAMRA-3′; β-actin forward, 5′-TGGTGATGGAGGAGGTTTAGTAAGT-3′ and reverse, 5′-AACCAATAAAACCTACTCCTCCCTTAA-3′, with the TaqMan probe 5′-6FAM-ACCACCACCCAACACACAATAACAAACACA-TAMRA-3′. The housekeeping β-actin gene (ACTB) was used to normalize a methylation independent control reaction. For relative quantification, amounts of the methylated DNA (percentage of methylated reference) at a β-catenin promoter region were normalized to the methylation value of the calibrator, which was defined as 100%. Universal methylated DNA (QIAGEN) was used as the calibrator. The percentage of methylated reference was defined as 100×2^(sampleACTB(ct) − sampleβ-catenin (ct))^/2^(calibratorACTB(ct) − calibratorβ-catenin (ct))^ [20]. To discriminate between individual methylation levels, a cutoff value of 4% or greater was defined as “methylated” and a cutoff value of less than 4% as “unmethylated” based on a previous study [21].

### Bisulfite sequencing PCR (BSP) of β-catenin promoters

We used the Methyl Primer Express v1.0 software (Applied Biosystems, Foster City, CA, USA) to analyze the *CTNNB1* gene promoter region -1,124–11,114 bp. DNA from lung cancer cells was extracted and then treated with sodium bisulfite using an EZ DNA methylation kit (Zymo Research) according to the manufacturer's instructions. The β-catenin promoter-specific primers for bisulfite sequencing were constructed using promoter sequence data and primer design software (Primer Premier 5; Premier Biosoft International, Palo Alto, CA, USA); the primer sequences are shown in Table 1. The primers were designed to amplify a CpG-rich region of the promoter spanning 189 CpG sites (19 CpGCpG sites). The PCR products were purified using the multifunctional DNA purification extraction kit (DP1501, BioTeke Corporation, Beijing, China) and ligated into the pUM-T simple vector (DP6803, BioTeke Corporation, Beijing, China), and at least five separate clones each were chosen for sequence analysis by BiQ Analyzer.

**Table 1:** Primer sequences of BSP.

Name	Sequence (5′–3′)	Length	Tm	Size
1 F	TGCGATTTAGGTTTAGTAGGGAGTGT	26	62.4	345
1 R	AATATCCTCCCCTATCCCAAACC	24	62	
2 F	ATAGGGGAGGATATTAGGGTTATT	24	56.7	336
2 R	TAACGCCGCACAAAAAACTCTTAT	24	62.7	
3-1 F	GGAGGAAGGTTTGAGGAGTAGTTTTAG	27	62.3	387
3-1 R	CCGCCTACCATCCG/AACTCCTATA	24	65.7	
3-2 F	TATAGGAGTTCGGATGGTAGG	21	54	232
3-2 R	CCCCAAAACTAATAAAACTTAAAATAAC	28	58.8	
3-3 F	CGGCGTTATTTTAAGTTTTTCG	22	59.5	104
3-3 R	AAACTACTCCTCAAACCTTCCT	22	53.9	

### Treatment with 5-aza-dC and Wnt3a

Cultivated lung cancer cells were treated with different concentrations of 5-aza-2′-deoxycytidine (5-aza-dC) (Sigma) dissolved in culture medium every 24 hours. Using q-MSP and MTT assays, the drug concentration required for β-catenin promoter CpG island demethylation with no significant effect on cell growth was selected (7 µM) and cells were collected after continued culture for 48 hours (Figure S1).

Cells were treated with 500 ng/ml recombinant Wnt3a (R&D Systems, Minneapolis, MN, USA) for 48 hours.

#### Small interfering RNA treatment

Cell lines were plated in six-well plates with fresh media without antibiotics for 24 h before transfection. Cells were transfected with β-catenin siRNA (sc-29209, Santa Cruz Biotechnology, Santa Cruz, CA, USA), E-cadherin siRNA (sc-35242, Santa Cruz) and control siRNA (sc-37007, Santa Cruz Biotechnology), using Lipofectamine-2000 (Invitrogen) according to the manufacturer's instruction. All the siRNAs were composed of a pool of two target-specific 19–25 nt siRNAs, The cells were harvested at 48 hours post-transfection.

#### Western blot analysis

Total protein from cells was extracted in lysis buffer (Pierce) and quantified using the Bradford method. Then, proteins (50 µg) were separated by SDS-PAGE (12%) and transferred to polyvinylidene fluoride (PVDF) membrane (Millipore, Billerica, MA, USA). Subsequently, membranes were incubated overnight at 4°C with monoclonal antibodies against E-cadherin (sc-8426, 1:200; Santa Cruz Biotechnology), β-catenin (610154, 1:500; BD Transduction Laboratories, Lexington, KY, USA) or GAPDH (sc-365062, 1:500, Santa Cruz Biotechnology). After incubation with peroxidase coupled anti-mouse IgG (Santa Cruz Biotechnology) at 37°C for 2 h, bound proteins were visualized using ECL (Pierce) and detected using BioImaging Systems (UVP Inc., Upland, CA, USA). The relative protein levels were calculated based on GAPDH protein as a loading control.

#### Matrigel invasion assay

Cell invasion assays were performed using a 24-well transwell chamber with a pore size of 8 µm (Corning Costar Transwell, Cambridge, MA, USA), and the inserts were coated with 20 µl Matrigel (1∶3 dilution, BD Biosciences). Then, 48 h after transfection, cells were trypsinized and transferred to the upper Matrigel chamber in 100 µl of serum-free medium containing 3×10^5^ cells and incubated for 16 hours. Medium supplemented with 10% FBS was added to the lower chamber as the chemoattractant. The numbers of invading cells were counted in 10 randomly selected high-power microscope fields. The experiments were performed in triplicate.

#### Immunofluorescent staining

The cells were fixed with 4% paraformaldehyde, blocked with 1% BSA and then incubated with the β-catenin monoclonal antibody (610154, 1:400; BD Transduction Laboratories) overnight at 4°C, followed by incubation with fluorescein isothiocyanate (FITC) conjugated secondary antibodies (1:200, Zhongshan Golden Bridge, Beijing, China) at 37°C. The nuclei were counterstained with 4′, 6-diamidino-2-phenylindole (DAPI). Epifluorescent microscopy was performed using an inverted Nikon TE300 microscope (Melville, NY, USA) and confocal microscopy was performed using a Radiance 2000 laser scanning confocal microscope (Carl Zeiss, Thornwood, NY, USA).

#### Dual-luciferase assays

Transfections were performed using Lipofectamine 2000 (Invitrogen) according to the manufacturer's instructions. Cells were plated in 24-well plates for 24 h prior to transfection with pGL3-OT or pGL3-OF (0.5 mg) reporter gene plasmids, together with the control plasmid pRL-TK (50 ng). After incubation for 30 h at 37°C, reporter gene expression was detected by the Dual-Luciferase Assay System (Promega, Madison, WI, USA). Tcf-mediated gene transcription activity was determined by the ratio of pGL3-OT to pGL3-OF luciferase activity, which was normalized to Renilla luciferase activity from the control plasmid pRL-TK. All experiments were performed, in duplicate, a minimum of three times. To analyze the effects of 5-aza-dC and β-catenin siRNA on Wnt signaling, 5-aza-dC was added and β-catenin siRNA was co-transfected with pGL3-OT or pGL3-OF for luciferase assays.

### Immunohistochemistry (IHC)

Immunohistochemical analysis of β-catenin expression was performed using a β-catenin-specific monoclonal antibody (610154, 1:200; BD Transduction Laboratories) as previously described and was evaluated according to our previous report [17], [22].

#### Statistical analysis

SPSS version 13.0 for Windows (SPSS Inc., Chicago, IL, USA) was used for all analyses. The chi-square and Spearman's correlation tests were used to examine possible correlations of β-catenin methylation status in NSCLC with its methylation status in adjacent normal lung tissue and clinicopathologic factors. The Mann-Whitney U-test was used to analyze the results of q-MSP, Western blot, Matrigel invasive assays and Dual-luciferase assays. The Kaplan-Meier method was used to estimate the probability of patient survival, and differences in the survival of subgroups of patients were compared using Mantel's log-rank test. Data were presented as the mean of experiments performed in triplicate. *P*<0.05 was considered to indicate statistically significant differences.

## Results

### Analysis of the methylation status of the promoter region of the β-catenin gene in lung cancer cells

**Figure 1:**
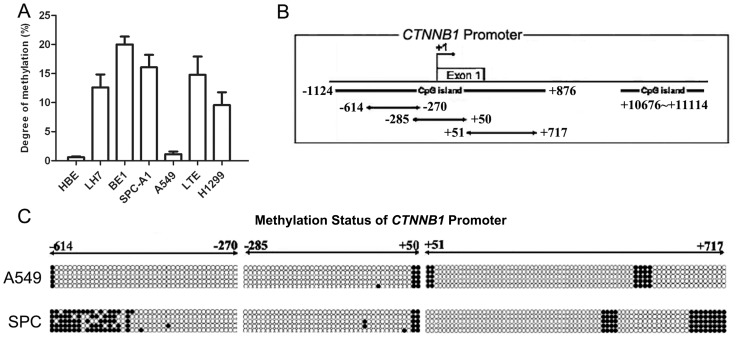
Analysis of methylation status of β-catenin promoter in NSCLC cell lines. (A) Quantitative MSP analysis of the β-catenin promoter region in NSCLC cell lines. (B) Promoter region of β-catenin gene: Position of CpG islands and primer design are indicated by lines and arrows. (C) Methylation of one of the 19 CG-dinucleotides in region −614 bp to −270 bp of both A549 and SPC. Filled circles represent methylated CG sites, unfilled circles represent unmethylated CG sites.

The β-catenin promoter methylation levels were tested in lung cancer cell lines. As shown in Figure 1A, five cell lines (LH7, BE1, SPC, LTE and H1299) exhibited high levels of methylation, while two cell lines (HBE and A549) were unmethylated. Analysis of the CTNNB1 gene promoter region −1,124 – 11,114 bp in NSCLC cell lines revealed the presence of two CpG islands at positions −1,124 – 876 and 10,676 – 11,114, respectively. These sequences contained 189 single CG sites, 19 of which were CG-dinucleotides. Using the sodium bisulfite genomic sequencing method, we designed primers to analyze the first CpG islands in a 1,331 base pair region (−614–717) containing part of the β-catenin promoter region across its transcriptional start site in the SPC and A549 cell lines. Multiple methylation sites were identified in SPC cells, whereas only a few 5-methylcytosine residues were observed in several sequenced clones derived from A549 cells (Fig. 1B and 1C).

### Treating cells with 5-aza-dC restored β-catenin expression and inhibited lung cancer cell invasiveness through re-establishing membranous β-catenin expression rather than by activation of the Wnt signaling pathway

**Figure 2:**
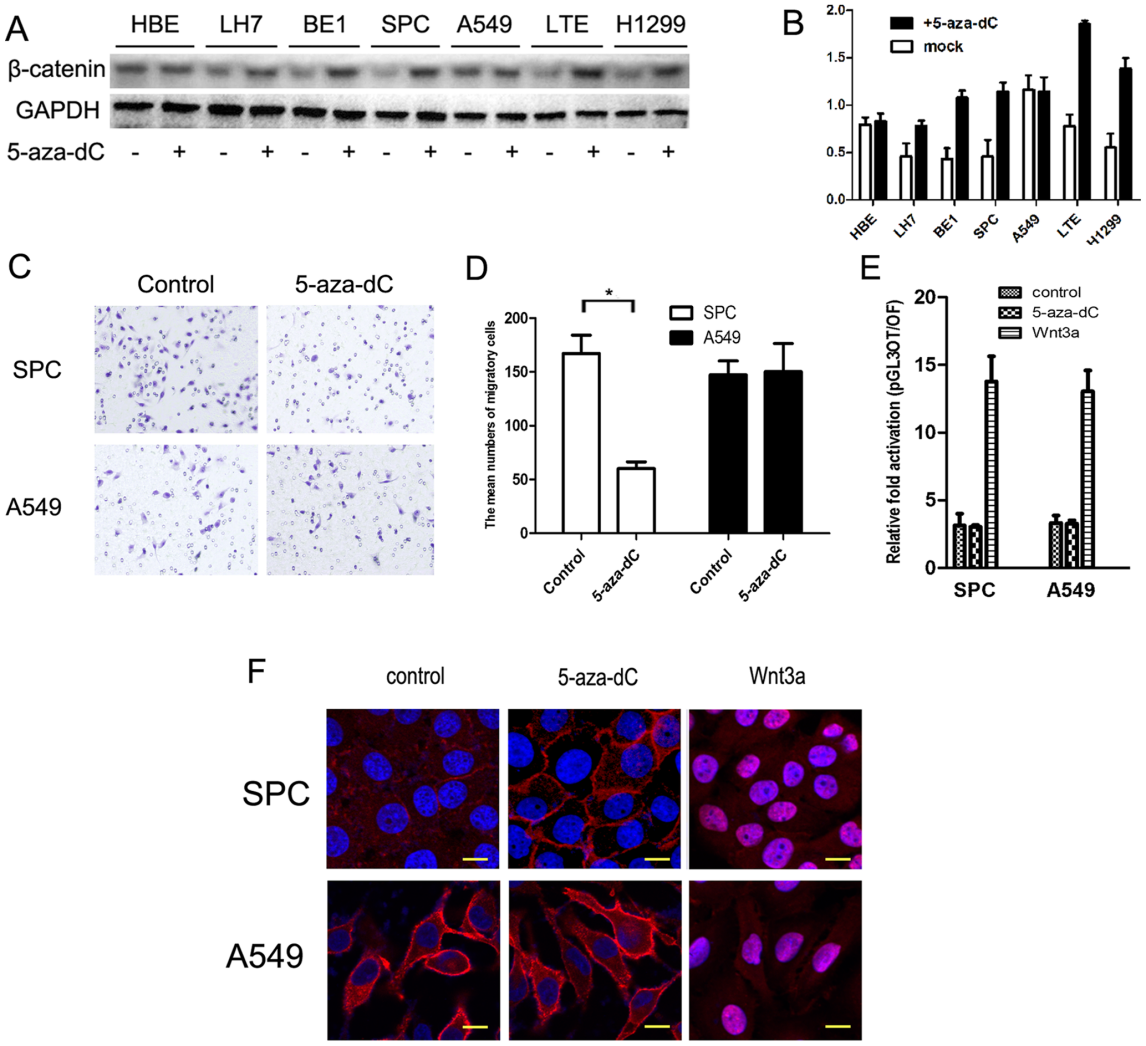
Effects of 5-aza-dC treatment in SPC and A549. (A) Protein levels of β-catenin after 5-aza-dC treatment (7 µM) in NSCLC cell lines. (B) Quantification of protein expression after 5-aza-dC treatment. (C) Representative images of cells on the bottom of the Transwell membranes show the changes in invasive cell numbers (×400, Scale bar = 50 µm). (D) Number of cells invading onto the lower surface of the filter was counted, each carried out in triplicate. (Bars represent SD. *P<0.05, compared to the control). (E) Dual-luciferase assay results show that 5-aza-dC treatment resulted in no significant changes in Wnt signaling activities compared with Wnt3a treated cells. Each of the experiments was repeated in triplicate. (D) Immunofluorescent staining showing that β-catenin is primarily localized at the membrane in SPC and A549 cell lines. Treatment with 5-aza-dC altered the amounts of membranous β-catenin expression. However, β-catenin was translocated into the nucleus following Wnt3a treatment (Scale bar = 20 µm, β-catenin antibody was replaced by normal mouse IgG as a negative control).

The chemical 5-aza-dC has been used both in vitro and in vivo to inhibit DNA methylation. In this study, all seven cell lines (HBE, LH7, BE1, SPC, A549, LTE and H1299) were treated with 5-aza-dC (Fig. 2A, 2B). Expression of β-catenin was restored in the five cell lines (LH7, BE1, SPC, LTE and H1299) in which β-catenin promoter methylation had been confirmed previously. However, no significant changes were seen in the HBE and A549 cell lines (unmethylated). We further investigated the effects of 5-aza-dC treatment on the invasion capacity of lung cell lines. As shown in Figures 2C and 2D, 5-aza-dC treatment resulted in marked inhibition of invasiveness in SPC cells, while there was no measurable effect in A549 cells (See Figure S2 for matrigel results in other cell lines). Subsequently, the capability of 5-aza-dC to activate the Wnt signaling pathway was investigated using Wnt3a protein-simulated cells as a positive control. Compared with Wnt3a-treated cells, dual-luciferase assays revealed that 5-aza-dC treatment resulted in no significant changes in Wnt signaling activities (Fig. 2E). Immunofluorescence analysis revealed that treatment with 5-aza-dC restored membranous β-catenin expression in SPC, but not A549 cells. Furthermore, β-catenin expression was detected in the nucleus in Wnt3a-treated cells, whereas, a membranous distribution was observed in untreated cells (Fig. 2F). Taken together, these data suggested that, unlike in other carcinomas, methylation of β-catenin promoter was a common phenomenon in NSCLC, and may be responsible for the downregulation of β-catenin expression enhancing the invasiveness of lung cancer cell.

### 5-Aza-dC treatment restored β-catenin expression in an E-cadherin independent manner

**Figure 3:**
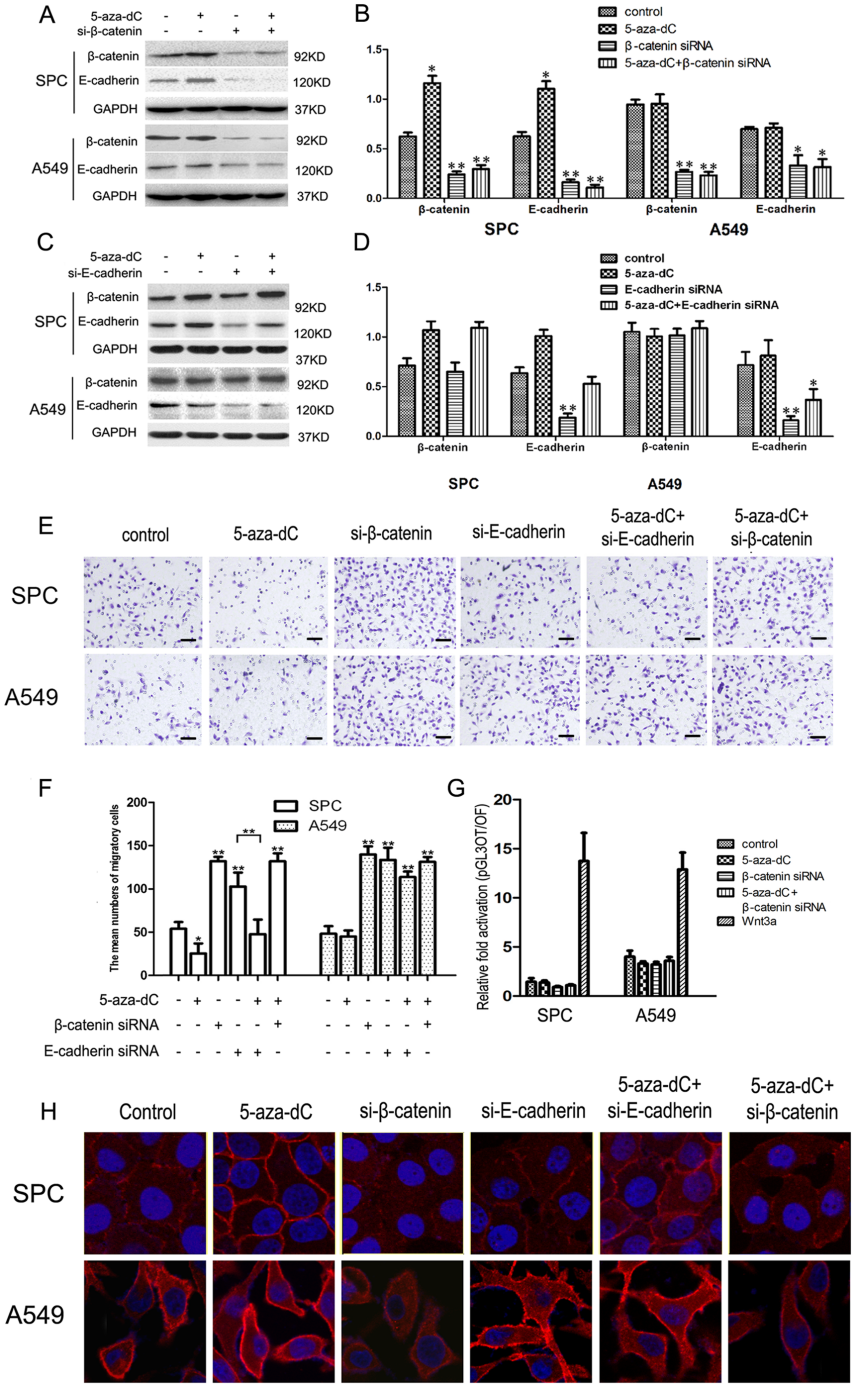
Effect of 5-aza-dC treatment with β-catenin and/or E-cadherin depletion. After knockdown of β-catenin (A) or E-cadherin (C), protein expression of SPC and A549 cell lines were examined 48 h after 5-aza-dC treatment using the indicated antibodies. (B, D) Graphs show the quantification of protein expression after 5-aza-dC treatment and siRNA transfection. GAPDH was used as a control. (E) Representative images of cells on the bottom of the Transwell membranes show the changes in invasive cell numbers (×400, Scale bar = 50 µm). (F) Number of cells invading onto the lower surface of the filter was counted, each carried out in triplicate. (Bars represent SD. *P<0.05, compared to the control). (G) Dual-luciferase assay results show that both 5-aza-dC treatment and β-catenin depletion resulted in no significant changes in Wnt signaling activities compared with Wnt3a treated cells. Each of the experiments was repeated in triplicate. (H) Immunofluorescent staining showing that the membranous β-catenin is enhanced by 5-aza-dC treatment only in SPC cell line, but not in A549 cell line. Depletion of β-catenin attenuates membranous β-catenin in both SPC and A549 cell lines, but depletion of E-cadherin has no effect on membranous expression of β-catenin in either SPC or A549 cell line. Treatment with 5-aza-dC slightly altered the amounts of membranous β-catenin expression after β-catenin or E-cadherin depletion in SPC cell line but not in A549 cell line. (Scale bar = 20 µm, β-catenin antibody was replaced by normal mouse IgG as a negative control).

In order to further confirm the function of promoter methylation of β-catenin, β-catenin-specific siRNA was introduced into SPC and A549 cells. Compared with cells transfected with control scrambled siRNA, cells transfected with β-catenin siRNA exhibited reduced β-catenin and E-cadherin expression, which cannot be restored by 5-aza-dC treatment (Fig. 3A, B). Next, we knocked down E-cadherin with E-cadherin-specific siRNA in these two cell lines. E-cadherin expression was decreased in both SPC and A549 cells, whereas, β-catenin expression was not visibly decreased by E-cadherin depletion compared with the control. With 5-aza-dC treatment, β-catenin expression was significantly upregulated in SPC cells regardless the expression of E-cadherin (Fig. 3C, D). We further investigated the effects of 5-aza-dC treatment when introduced with β-catenin-siRNA and E-cadherin-siRNA on the invasion capacity of lung cell lines. As shown in Figures 3E and 3F, either β-catenin or E-cadherin depletion led to a significant increase in the invasive capacity of both SPC and A549 cells. By 5-aza-dC treatment, the number of the migratory cells was significantly decreased in E-cadherin-depleted SPC cells, which was not observed in A549 cells. However, the invasive capacities of β-catenin-depleted SPC and A549 cells were not obviously inhibited by 5-aza-dC treatment. Furthermore, dual-luciferase assays revealed that neither 5-aza-dC treatment nor β-catenin-depletion resulted in significant changes in Wnt signaling activities compared with Wnt3a-treated cells (Fig. 3G). Accordingly, the inhibition of β-catenin expression by its promoter methylation seems to downregulating the invasiveness of lung cancer cell in an E-cadherin independent manner. Immunofluorescent staining showed that the membranous β-catenin is enhanced by 5-aza-dC treatment only in SPC cell line, but not in A549 cell line. Depletion of β-catenin attenuates membranous β-catenin in both SPC and A549 cell lines, but depletion of E-cadherin has no effect on membranous expression of β-catenin in either SPC or A549 cell line. Treatment with 5-aza-dC slightly altered the amounts of membranous β-catenin expression after β-catenin or E-cadherin depletion in SPC cell line but not in A549 cell line.

### Hypermethylation of β-catenin promoter correlated with its loss of membranous expression in NSCLC sample

**Figure 4:**
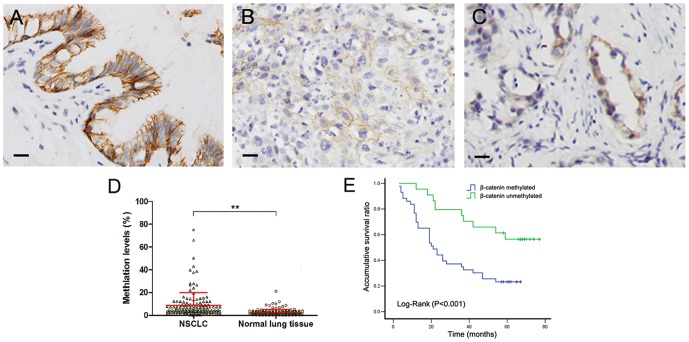
β-catenin expression and promoter methylation in normal lung tissues and lung cancer tissues. (A) β-catenin expression in normal lung epithelium with strong membranous staining. Membranous β-catenin expression was lost in lung squamous cell carcinoma (B) and lung adenocarcinoma (C). Scale bar = 20 µm. (D) Quantitative methylation results of q-MSP. Data represent mean±SD. The Mann-Whitney U-test was used to determine the statistical significance. **P<0.001. (E) Survival curves of patients with or without β-catenin promoter methylation. Mantel's log-rank test was used to determine statistical significance.

We next investigated the methylation status of β-catenin promoter in clinical tissue samples. β-Catenin was expressed predominantly on the cell membrane in normal lung tissue samples (83.9%, 120/143; Fig. 4A), with reduced membranous expression in NSCLC tissues (18.9%; 27/143; Fig. 4B, C). Subsequently, the β-catenin promoter methylation levels were quantitated by real-time methylation-specific PCR. Using a hypermethylation value of greater than 4% as a cutoff, hypermethylation of β-catenin (mean ± SD, 8.60%±11.41%; range, 0–100%) was found in 74 (51.7%) of the 143 NSCLC specimens, which was significantly higher than that in adjacent normal lung tissue (13.3%, 19/143; mean ± SD, 2.66%±2.63%; range, 0–100%; *P*<0.001; Fig. 4D). Taken together, our study suggested that β-catenin methylation significantly correlated with loss of membranous β-catenin expression (*P* = 0.001).

### The Correlation between β-catenin promoter methylation and clinicopathological factors

Clinical and pathological characteristics of patients with NSCLC were analyzed according to their methylation status. As shown in Table 2, β-catenin hypermethylation positively correlated with lymph node metastasis and TNM stage in the 143 cases of NSCLC (*P* = 0.001 and *P* = 0.046, respectively). There was no significant association between β-catenin methylation and sex, age, histological type, or differentiation in NSCLC. The Kaplan-Meier test revealed that the overall survival time of NSCLC patients was shorter in patients with β-catenin-methylated tumors than in those with the β-catenin-unmethylated tumors (*P*<0.001, Fig. 4E).

**Table 2:** Association between β-catenin gene methylation and clinicopathological features.

Item	Number	β-catenin methylation	χ^2^	*P* value	*r*
Gender					
Male	84	42	0.249	0.618	0.042
Female	59	32			
Age (years)					
<60	66	39	2.647	0.104	−0.136
≥60	77	35			
Histological type					
Squamous cell carcinoma	44	22	0.078	0.780	0.023
Adenocarcinoma	99	52			
Lymph node metastasis					
Negative	73	28	10.711	0.001	0.274
Positive	70	46			
Differentiation					
Well	51	23	1.404	0.236	−0.099
Moderately or poorly	92	51			
TNM classification					
I–II	81	36	3.991	0.046	0.167
IIIa–IIIb	62	38			

## Discussion

β-Catenin, as an important component of both adherens junctions and Wnt signaling pathway, was believed to be translocated into nucleus by its exon mutation and/or APC mutation in various tumor type except lung cancer [4], [7], [8]. However, accumulated evidences indicate that, in the lung cancer, β-catenin is lost and Wnt signaling activity is downregulated. The underlying mechanism remains unelucidated [5], [9]–[11]. Our most recent study found that the promoter of β-catenin gene was always methylated in lung cancer cell lines [16]. In order to clarify the functions of β-catenin in NSCLC, we expanded our investigation of the promoter methylation status in seven lung cancer cell lines. We found that the demethylation agent 5-aza-dC restored β-catenin protein levels in five out of seven cell lines that had been confirmed to be methylated in the present study. Among these, the methylation status of SPC and A549 was further confirmed by bisulfite genomic sequencing. It is well recognized that the entry of β-catenin to the nucleus could enhance Wnt signaling activity, thereby promoting the invasiveness of multiple human cancers. However,β-catenin expression was lost in distant metastases of gastric carcinoma and the loss of β-catenin expression may be due to hypermethylation of the β-catenin promoter, despite that β-catenin expression was upregulated and the Wnt signaling pathway was activated in most of the primary gastric cancer [15]. We speculated that loss of β-catenin may trigger other signaling cascade(s) stronger than Wnt signaling activation in controlling the invasiveness of cancer cells. Since lung cancer shows lower baseline of Wnt signaling activity than gastric cancer, the main mechanism in regulating invasiveness may be independent of Wnt signaling pathway. activation of Wnt signaling pathway, the invasion capacity, and the subcelluar distribution of β-catenin in lung cell lines. The results indicated that demethylation by 5-aza-dC could restored β-catenin expression and inhibited lung cancer cell invasiveness through re-establishing membranous β-catenin expression, rather than by the activation of the Wnt signaling pathway. The present study offered a new explanation for why β-catenin expression was downregulated in NSCLC and indicated that the promoter methylation of β-catenin may regulate invasiveness through a pathway independent of Wnt signaling in lung cancer cells. As catenins together with cadherins and formed adherens junctions, our results indicated that loss of β-catenin expression might destruct adherens junctions by its reduced membranous expression.

E-cadherin plays a crucial role in adherens junctions. Through its intracellular domain, E-cadherin was connected with β-catenin. Although the methylation of E-cadherin gene methylation was found in various cancers, it remains controversial as for in the lung cancer [23]–[25]. Russo *et al*. found that methylation of the E-cadherin gene seemed to occur only in the early stages of bronchial carcinogenesis [26]. Moreover, a recent report demonstrated that E-cadherin was rarely methylated in the lung cancer [27]. In order to eliminate the interference of E-cadherin gene methylation, we investigated the changes in the effects of demethylation agent following the introduction of E-cadherin siRNA or β-catenin siRNA into SPC and A549 cells. Western blotting results showed that demethylation treatment restored β-catenin expression only in SPC, which is formerly verified for β-catenin promoter methylation. Moreover, β-catenin restoration or depletion altered E-cadherin protein levels correspondingly, while β-catenin proteins were not affected by E-cadherin depletion. Since 5-aza-dC can restore the expression of E-cadherin only in SPC but not A549, we speculated that the restoration of E-cadherin could be attributed to the demethylation of β-catenin promoter and restoration of its expression. Matrigel invasion assay data revealed that both β-catenin depletion and E-cadherin depletion upregulated the invasiveness of SPC and A549 cells. However, following 5-aza-dC treatment of E-cadherin depleted cells, the invasiveness of SPC cells with β-catenin promoter hypermethylation was significantly downregulated. Surprisingly, immunofluorescence results showed that β-catenin was still localized on the membrane in both A549 and SPC cells after E-cadherin depletion. Furthermore, the membranous β-catenin expression was enhanced after treated with 5-aza-dC in SPC cells transfected with E-cadherin siRNA, but not in A549 cells. The present study indicated that β-catenin could regulate the stability of adherens junctions independent of E-cadherin. Our results are consistent with previous reports that E-cadherin is important for the establishment but not the maintenance of cell-cell contacts in some cell types [12], [28], [29]. Moreover, Kumper *et al.* found that silencing β-catenin could reduce E-cadherin levels butnot vise versa. They finally proved that the β-catenin combined with P-cadherin but not E-cadherin in prostate cancer cells. Similarly, based on our results, β-catenin may combined with other cadherins and form a complex independent of E-cadherin [12]. Although β-catenin have been reported to affect cadherin-mediated adhesion, and the depletion of β-catenin in an E-cadherin-deficent cell line led to a decrease in invasion which was thought to be attributed to the activation of Wnt signaling. Our results of dual-luciferase assays showed that 5-aza-dC treatment caused no significant increase in Wnt signaling activities in lung cancer cells [30]. Taken together, we speculated that E-cadherin is not the only cadherins that binding with β-catenin and maintains the integrity of adherens junctions. Therefore, extra studies are required to further elucidated and confirm the mechanisms in regulating adherens junctions in NSCLC.

The present study found that β-catenin promoter methylation is responsible for its loss of membranous expression. We investigated the methylation status of β-catenin promoter in clinical NSCLC samples. The hypermethylation of the β-catenin promoter positively correlated with clinicopathological factors, such as lymph node metastasis, TNM stage and poor patient survival in clinical NSCLC samples. Loss of β-catenin was reportedly correlated with lymph node metastasis, high TNM stages in multiple cancers and associated with poor prognosis in NSCLC [11], [31], [32]. Taken together, our results indicated that promoter methylation of β-catenin might be an important mechanism in the regulation of invasiveness and metastasis in lung cancer development.

In summary, our data indicates that β-catenin expression is frequently lost in NSCLC and that the loss of β-catenin expression may be due to hypermethylation of the β-catenin promoter. Metastases formation is dependent on the destruction of adherens junctions. Therefore, the reduced expression of β-catenin in NSCLC and its possible inactivation through hypermethylation of its promoter indicate a novel mechanism for the formation of metastasis and the progression of NSCLC. Hypermethylation of the β-catenin promoter appears to be a predictor of poor clinical outcomes in NSCLC patients. Although the demethylation agent, 5-aza-dC, acts on genomic DNAs as a whole, the results of our study indicate the potential of β-catenin as a new epigenetic target for the treatment of NSCLC patients.

## Supporting Information

Figure S1 **Selection of appropriate concentration for 5-aza-dC treatment.** After adding 0, 5, 7 and 10 µM 5-aza-dC into the medium for 48 hours, MSP (A) and MTT (B) were performed. The promoter of β-catenin was demethylated in cells treated with 5-aza-dC in concentration of 7 and 10 µM in both SPC and LTE cells (A). MTT results showed that 7 µM was the minimum concentration for demethylation with relatively small growth inhibition (Bars represent SD. *P<0.05 and ** *P*<0.01 compared to the 0-µM group).
(TIF)

Figure S2 **Effect of 5-aza-dC treatment in the other cell lines with methylations on the β-catenin promoter.** Matrigel assay was performed in LH7, BE1, LTE and H1299 cells to test the invading abilities of the cells upon 5-aza-dC treatment (A, ×400, Scale bar = 50 µm) Number of cells invading into the lower surface of the filter was counted, and each experiment was carried out in triplicates. (B, Bars represent SD. *P<0.05, compared to the control).
(TIF)
