# Supplementary figures and images for: Promoter Methylation-Mediated Silencing of β-Catenin Enhances Invasiveness of Non-Small Cell Lung Cancer and Predicts Adverse Prognosis
Source: PLoS One. 2014 Nov 14;9(11):e112258. doi: 10.1371/journal.pone.0112258 (PMC4232381; doi:10.1371/journal.pone.0112258)

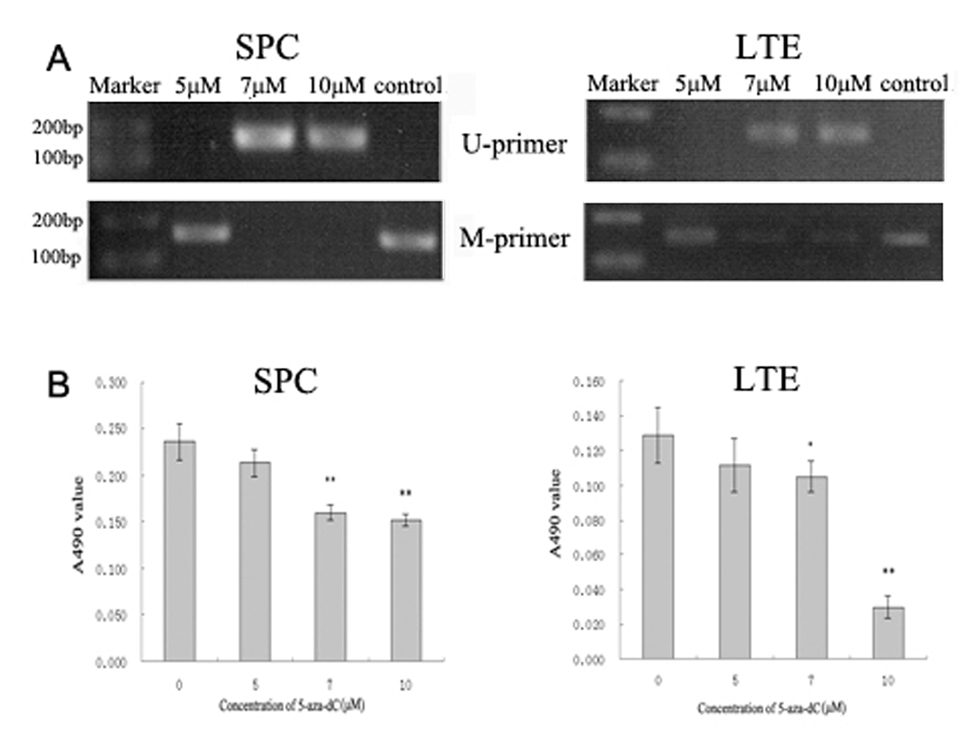

Supplement: Figure S1 — Selection of appropriate concentration for 5-aza-dC treatment. After adding 0, 5, 7 and 10 µM 5-aza-dC into the medium for 48 hours, MSP (A) and MTT (B) were performed. The promoter of β-catenin was demethylated in cells treated with 5-aza-dC in concentration of 7 and 10 µM in both SPC and LTE cells (A). MTT results showed that 7 µM was the minimum concentration for demethylation with relatively small growth inhibition (Bars represent SD. *P<0.05 and ** P<0.01 compared to the 0-µM group). (TIF) [file pone.0112258.s001.tif]

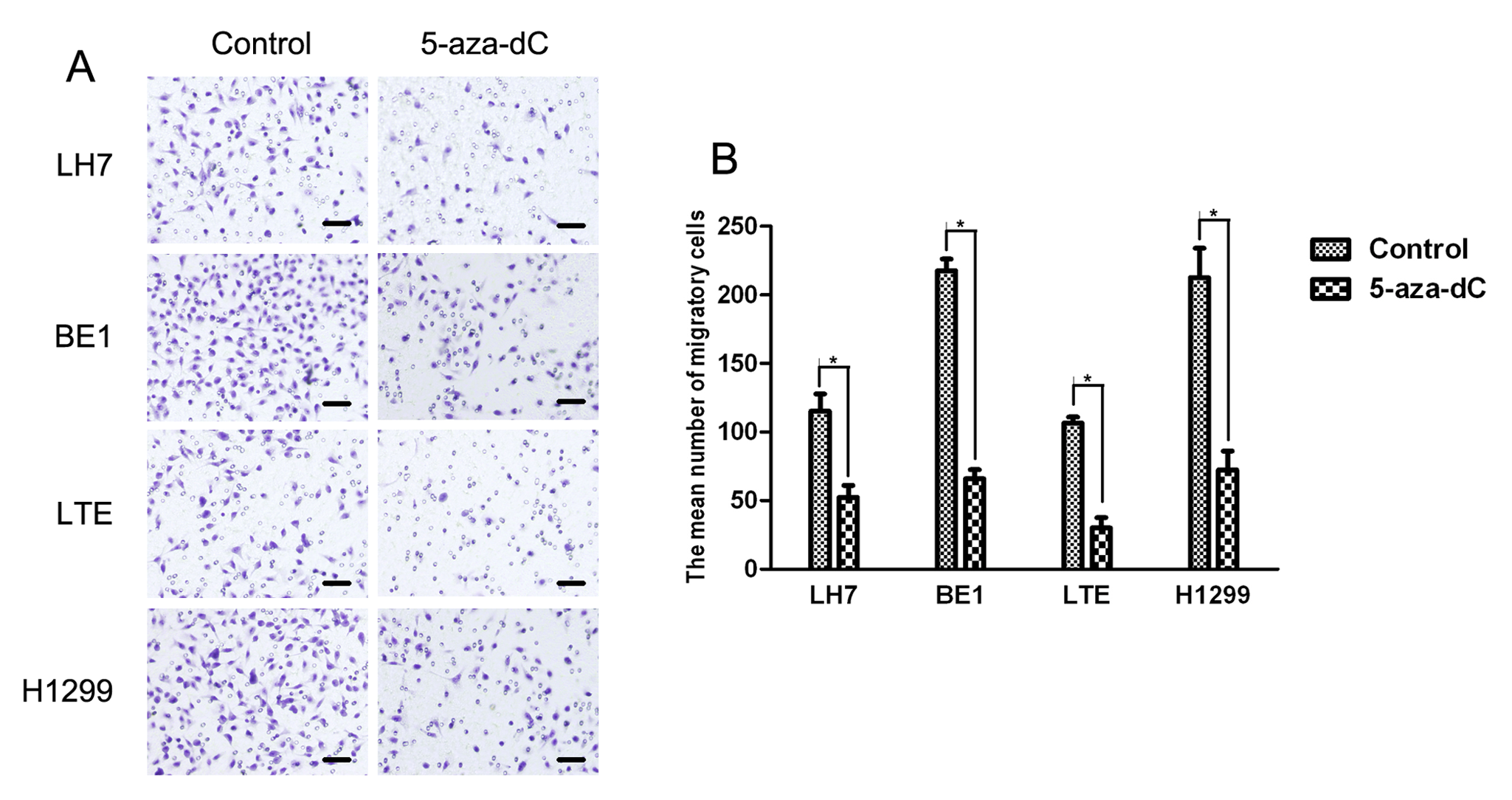

Supplement: Figure S2 — Effect of 5-aza-dC treatment in the other cell lines with methylations on the β-catenin promoter. Matrigel assay was performed in LH7, BE1, LTE and H1299 cells to test the invading abilities of the cells upon 5-aza-dC treatment (A, ×400, Scale bar = 50 µm) Number of cells invading into the lower surface of the filter was counted, and each experiment was carried out in triplicates. (B, Bars represent SD. *P<0.05, compared to the control). (TIF) [file pone.0112258.s002.tif]
